# Nanomolar Oxytocin Synergizes with Weak Electrical Afferent Stimulation to Activate the Locomotor CPG of the Rat Spinal Cord *In Vitro*

**DOI:** 10.1371/journal.pone.0092967

**Published:** 2014-03-21

**Authors:** Francesco Dose, Patrizia Zanon, Tamara Coslovich, Giuliano Taccola

**Affiliations:** 1 Neuroscience Department, International School for Advanced Studies (S.I.S.S.A.), Trieste, Italy; 2 Spinal Person Injury Neurorehabilitation Applied Laboratory (S.P.I.N.A.L.), Istituto di Medicina Fisica e Riabilitazione (IMFR), Udine, Italy; University of Toronto, Canada

## Abstract

Synergizing the effect of afferent fibre stimulation with pharmacological interventions is a desirable goal to trigger spinal locomotor activity, especially after injury. Thus, to better understand the mechanisms to optimize this process, we studied the role of the neuropeptide oxytocin (previously shown to stimulate locomotor networks) on network and motoneuron properties using the isolated neonatal rat spinal cord. On motoneurons oxytocin (1 nM–1 μM) generated sporadic bursts with superimposed firing and dose-dependent depolarization. No desensitization was observed despite repeated applications. Tetrodotoxin completely blocked the effects of oxytocin, demonstrating the network origin of the responses. Recording motoneuron pool activity from lumbar ventral roots showed oxytocin mediated depolarization with synchronous bursts, and depression of reflex responses in a stimulus and peptide-concentration dependent fashion. Disinhibited bursting caused by strychnine and bicuculline was accelerated by oxytocin whose action was blocked by the oxytocin antagonist atosiban. Fictive locomotion appeared when subthreshold concentrations of NMDA plus 5HT were coapplied with oxytocin, an effect prevented after 24 h incubation with the inhibitor of 5HT synthesis, PCPA. When fictive locomotion was fully manifested, oxytocin did not change periodicity, although cycle amplitude became smaller. A novel protocol of electrical stimulation based on noisy waveforms and applied to one dorsal root evoked stereotypic fictive locomotion. Whenever the stimulus intensity was subthreshold, low doses of oxytocin triggered fictive locomotion although oxytocin *per se* did not affect primary afferent depolarization evoked by dorsal root pulses. Among the several functional targets for the action of oxytocin at lumbar spinal cord level, the present results highlight how small concentrations of this peptide could bring spinal networks to threshold for fictive locomotion in combination with other protocols, and delineate the use of oxytocin to strengthen the efficiency of electrical stimulation to activate locomotor circuits.

## Introduction

It is well known that the thoraco-lumbar spinal cord of mammals contains the neuronal hardware, indicated as central pattern generator (CPG), required to express the basic program that drives the alternated activation of flexor and extensor limb muscles during gait [1], [2]. The locomotor output is already present at birth and depends on the biophysical properties of motoneurons and interneurons composing the CPG, as well as on the connectivity among the elements of the network [3].

Neuromodulatory substances sculpt the rhythmic CPG pattern and confer the necessary flexibility to the network in response to demands from the external environment and afferent inputs [4]. Among the wide family of neuromodulators, certain agents can trigger locomotion, while others can speed it up or facilitate it in concomitance with suitable stimuli [4]. Drugs in the latter category are the most interesting, as they might be used to synergize rehabilitation techniques that exploit the proprioceptive physiological feedback [5], [6] to restore post-lesion locomotor patterns [7], [8], [9], [10], [11], [12]. Unfortunately, the drugs tested so far have shown contrasting results, underpinning the need for more efficient conjoint strategies, using both afferent and pharmacological stimulations [13]. However, *in vitro* studies have shown that neuromodulators can differently affect the chemically and electrically evoked fictive locomotion (FL) [14], [15], [16], indicating the complexity of network targets.

The neuropeptide oxytocin is a nona-peptide endogenously synthesized in the central nervous system, at the level of hypothalamic nuclei, medial amygdale, locus coeruleus and olfactory bulb [17]. In the spinal cord, oxytocin is exclusively localized within axons [18] and the majority of oxytocin-containing fibers originate from the hypothalamic paraventricular nucleus (PVN) [19], as confirmed, in the rat, by the complete disappearance of oxytocin after lesioning the PVN [20], [21]. Moderate presence of oxytocin-containing fibers (but not cell bodies) was confirmed in all laminae of the rat spinal cord, with clear predominance in laminae I, II, VII and X [22], [23] especially at lumbar level [18].

Oxytocin, which during neonatal life plays a role as trophic or differentiating factor during spinal cord maturation [23], [24], serves as a neurotransmitter on receptors coupled to different G proteins to mobilize intracellular Ca^2+^ and either open a non-specific cationic channel or close a K^+^ channel [25]. The neuronal distribution of oxytocin receptors (OTRs) parallels the distribution of its fibers [26], [27]. Oxytocin, on par with other neuropeptides, does not seem to work directly on its target, but rather it appears to have a neuromodulatory action in making it more responsive to any incoming inputs [28]. Endogenous oxytocin concentrations in the rodent cerebrospinal fluid (CSF) range from 15 to 80 pg/mL [29], that is similar to values of the human neonatal CSF (20–30 pg/mL) [30]. In the spinal cord, the overall content of oxytocin is rather homogeneous (< 70 pg/mm of tissue), although three times more oxytocin has been found in the first lumbar segments [31], where the locomotor CPG is mainly localized [2]. Nonetheless, there are only few studies about oxytocin role in the chemically-evoked locomotor network activity in vitro [32], [33]. Thus, there are no data on the effects of oxytocin in integrating afferent inputs into the CPG. The role of primary afferents in modulating the locomotor pattern is linked to the existence of sensory feedbacks evoked during gait to physiologically control, at a pre-synaptic level, incoming inputs to the spinal cord [34], and to convey facilitatory signals to the CPG via multisegmental sacrocaudal afferents [35], even with nociceptive content [36].

An innovative protocol of electrical stimulation, characterized by noisy waveforms and named FL*i*stim (Fictive Locomotion-induced stimulation) [37], has recently demonstrated to generate locomotor-like oscillations when delivered to a dorsal root (DR) or to sacrocaudal afferents of the isolated spinal cord.

Compared to classic protocols of electrical stimulation, which use trains of standard rectangular impulses [38], FL*i*stim requires a much lower stimulation strength and induces locomotor-like oscillations of longer duration [37]. Furthermore, FL*i*stim, as opposed to trains of pulses traditionally delivered to dorsal afferents, synergizes with sub-threshold concentrations of N-methyl-D-aspartate (NMDA) and serotonin (5-hydroxytryptamine, 5HT) to activate the CPG [39]. Using the *in vitro* rat spinal cord, the present study aims at exploring whether the neuropeptide could facilitate the effects of FL*i*stims, comparing it with chemically-evoked FL, and relating to its actions of synaptic transmission, network rhythmicity induced by pharmacological disinhibition [40] and motoneuron properties.

## Methods

### Spinal cord preparation and electrophysiological recordings

All experiments involving the use of rats and the procedures followed therein were approved by the Scuola Internazionale Superiore di Studi Avanzati (SISSA) ethics committee and are in accordance with the European Union guidelines. Animals were maintained in accordance with the guidelines of the Italian Animal Welfare Act. Spinal cords of neonatal Wistar rats (0 – 5 days old) were isolated from the mid-thoracic segments to the *cauda equine*, as previously described [41]. All efforts were made to minimize number and suffering of animals used for the experiments.

After surgical dissection, each spinal cord was mounted in a small recording chamber maintained at a constant room temperature of 22°C and continuously superfused (5 mL/min) with oxygenated (95% O_2_ and 5% CO_2_) Krebs solution, composed as follows (in mM): 113 NaCl, 4.5 KCl, 1 MgCl_2_ 7H_2_O, 2 CaCl_2_, 1 NaH_2_PO_4_, 25 NaHCO_3_, and 11 glucose, pH 7.4.

For intracellular recordings, antidromically identified lumbar (L4 or L5) motoneurons [42] were impaled using microelectrodes filled with 3 M-KCl (30 – 40 MΩ resistance), in current-clamp conditions. The input resistance of motoneurons was obtained by delivering steps of current (amplitude from −0.8 to 0.8 nA, duration  =  80 ms). Current/voltage plots were linear within the voltage range recorded and their slope indicates cell input resistance. In control conditions, baseline input resistance and membrane potential of motoneurons were, on average, 47.73±19.50 MΩ (from 24.30 MΩ to 72.03 MΩ) and −65.72±6.15 (n = 16), respectively.

Nerve recordings were performed in DC mode, using tight-fitting suction electrodes, from the lumbar (L) ventral roots (VRs). As a routine, recordings were obtained from the left (l) and right (r) L2 VRs, which mainly convey flexor motor-pool signals to hindlimb muscles, and from the l and r L5 VRs, principally expressing extensor commands to the same hindlimbs [43].

Therefore, the characteristic alternation among the discharges recorded from the flexor and extensor motor pools and between the left and right sides of the cord proves activation of the locomotor CPG. Signals were recorded, digitized and analyzed adopting pClamp software (version 10.3; Molecular Devices, PA, USA).

FL rhythm is elicited by the continuous bath application of NMDA (1,5–5 μM; Tocris, Bristol, UK) plus 5HT (4–10 μM; Sigma, Milan, Italy). Subthreshold pharmacological stimulation is obtained by reducing the concentration of NMDA + 5HT to the minimum required to induce a stable FL rhythm. To reduce the synthesis of endogenous 5HT, several experiments were performed where spinal cords were maintained overnight in Krebs solution containing the tryptophan hydroxylase inhibitor, p-chlorophenylalanine (PCPA, 10 μM; Sigma, Milan, Italy) in accordance with Branchereau et al. [44]. On the following day we recorded, in the continuous presence of PCPA, FL evoked by NMDA (5 μM) plus 5HT (10 μM). Afterwards, oxytocin (100 nM or 1 μM) was added to subthreshold concentrations of neurochemicals as indicated earlier. Control sham preparations were kept for the same period in Krebs solution, to confirm that maintaining the spinal cord for 1 day in vitro does not change the characteristics of chemically induced FL [45]. Longer in vitro maintenance was not viable, since after 2 days in vitro, only 1 out of 4 cords could express a brief episode of FL induced by NMDA (5 μM) and 5HT (10 μM).

Out of a series of 19 experiments, we considered for analysis only those which expressed stable FL after 10 min of continuous superfusion with NMDA (5 μM) + 5HT (10 μM): that is, 6 cords in Krebs solution and 9 in the group treated with PCPA (10 μM), respectively.

A disinhibited bursting, that was synchronous among all VRs, was produced by the pharmacological blockage of spinal inhibition, mediated by GABA_A_ and glycine receptors, with a continuous bath-application of strychnine (1 μM; Tocris, Milano, Italia) and bicuculline methiodide (20 μM; Abcam PLC, Cambridge, UK). To validate the specificity of OTR activation, atosiban was used at the same concentration that showed a selective antagonism on *in vitro* experimental preparations of neonatal rat central nervous system (5 μM) [46]. To reduce synaptic input on motoneurons, the broad sodium channels blocker, TTX (Abcam PLC, Cambridge, UK), was applied at the concentration of 1 μM until electrically evoked antidromic spikes fully disappeared (5 min). Afterwards, TTX was continuously perfused at lower concentration (250 nM) to maintain sodium current block throughout the experiment [47].

### Single or repetitive electrical stimulation of dorsal or ventral roots

Single electrical pulses were applied to either DRs or VRs via a bipolar suction electrode, in order to evoke either DR-DR potentials (DR-DRPs) [48], DR-VR potentials from the homologous VR (DR-VRPs) or antidromic action potentials from single motoneuron, respectively. Stimulus intensity was then calculated in terms of threshold (Th = 11.37±5.63 μA), which is defined as the minimum intensity required to elicit a detectable response from the homologous VR.

The stimulating protocol FL*i*stim (Fictive Locomotion-*induced* stimulation) used corresponds to a 60 s segment of a chemically induced FL recorded in AC mode from a VR, that was randomly selected (range 0.1 Hz – 10 000 Hz; sampling rate  =  500 Hz). Through off-line analysis with Origin 9.0 software (OriginLab, North Hampton, MA), the maximum current amplitude was adjusted to pre-selected values, then the trace was exported (as an ASCII text file) to a programmable stimulator (STG 4002; Multi Channel Systems, Reutlingen, Germany). At last, FL*i*stim was delivered to one DR, using a bipolar suction electrode, at an optimal amplitude comprised within the range of 0.144±0.077×Th. On the other hand, adopted intensities for electrical subthreshold stimulation were considered equal to 0.095±0.069×Th.

### Analysis of rhythmic activity

Each FL rhythm was analyzed in terms of periodicity (considered as the time between the onset of two consecutive cycles) and amplitude (defined as the height of signals, expressed in μV, calculated from the baseline at the beginning of each cycle to its peak). Furthermore, regularity of rhythmic patterns was expressed by the period coefficient of variation (CV; displayed as standard deviation [SD] mean^−1^). The strength of coupling among pairs of VRs signals was defined by the cross-correlation function (CCF) analysis. A CCF greater than + 0.5 indicates that two roots are synchronous, while a CCF smaller than − 0.5 shows full alternation [49].

All parameters used for the definition of disinhibited bursting and its measures (duration, cycle period, number and frequency of intraburst oscillations) are in accordance with Bracci et al. [40]. Within each preparation and for each test conditions, at least 20 cycles of bursting were analyzed to average data.

All data are expressed as mean ± S.D., where “n” indicates the number of experiments. Before assessing statistical differences among groups, a normality test was performed to select the use of either parametric or non-parametric tests. Different statistical approaches were used to compare sets of data. For parametric values, the Student's t-test (paired or unpaired) was used for comparison between two groups of data and ANOVA (analysis of variance), followed by post hoc analysis with Dunnett's or Tukey method for more than two groups. When referring to non-parametric values, the tests used were Mann-Whitney or Wilcoxon for comparing two groups and Kruskal-Wallis ANOVA on ranks, followed by post hoc analysis with Dunn's method, for a number of groups greater than two. As for parametric data, one way repeated ANOVA measures were used when each one of the cords analyzed was exposed to more than two treatments. To assess the success rates in inducing an episode of alternating oscillations by weak FL*i*stims, plus increasing concentrations of oxytocin, with respect to weak FL*i*stim alone, we applied the chi-squared test. Collected results were considered significant when P<0.05.

## Results

### Nanomolar concentrations of oxytocin excite spinal motoneurons

**Figure 1:**
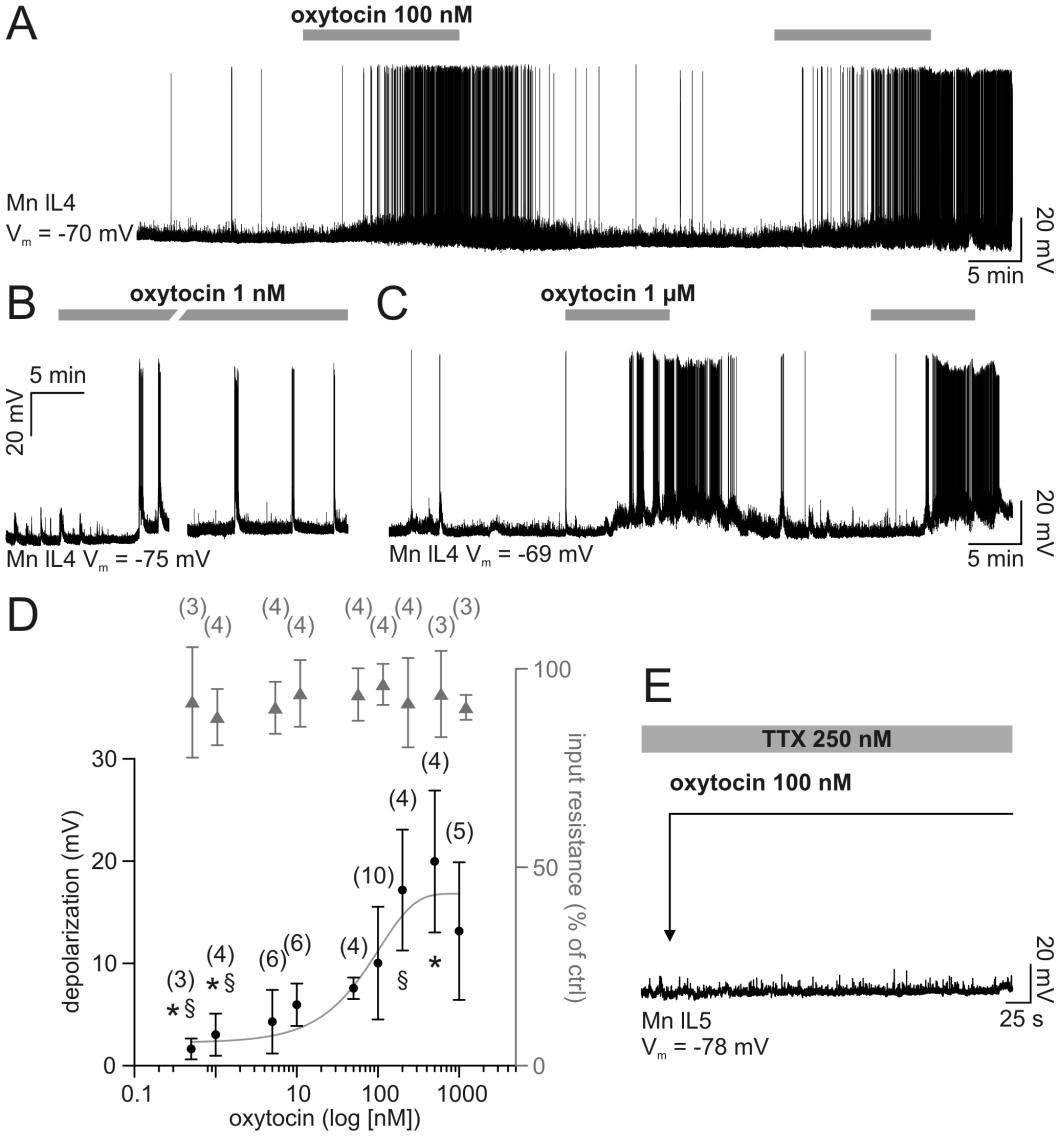
Oxytocin indirectly depolarizes single motoneurons. A, intracellular recording from a single motoneuron (lL4) shows that, after 5 min, oxytocin (100 nM; see gray bars) depolarises membrane potential and evokes high frequency spiking. Initial resting potential (V_m_) is −70 mV. B, lower concentration of oxytocin (1 nM; see bar) determines a slower (8 min) onset of bursts with intense firing activity despite minimal baseline depolarization that persists throughout the long neuropeptide perfusion (40 min). Initial resting potential is −75 mV. Note that the 10 min trace break corresponds to the time spent in generating tests for the cell I/V curve. Different cell from A. C, two consecutive applications of oxytocin (1 μM) induce reproducible responses when timed 20 min apart. Initial resting potential is −69 mV. Different cell from A, B. D, dose response plots of membrane potential depolarization (from baseline; fitted with sigmoidal curve; filled circles) and input resistance (as percentage value with respect to control; gray triangles) for cumulative doses of oxytocin (log scale). Symbols *, § indicate significant difference versus the higher concentrations data (Kruskal-Wallis one way ANOVA on ranks followed by all pairwise multiple comparison with Dunn's method; P<0.001; the number of preparations used to calculate the mean is shown in parentheses; the error bars indicate SD). E, sample trace from a single motoneuron (lL5) demonstrates that oxytocin (100 nM) fails to depolarize the cell when applied (see arrow) in the presence of network block by TTX (250 nM; gray bar).

The example of Fig. 1 A, recorded from a motoneuron under current clamp conditions, shows that, after 5 min application, oxytocin (100 nM) depolarized the cell (plateau amplitude  =  8.85 mV), with superimposed firing activity (2.14 Hz). The effects of oxytocin waned after 30 min washout when a second exposure to oxytocin induced similar depolarization (8.76 mV) and firing (2.9 Hz). A longer perfusion (40 min) at a lower concentration (1 nM, Fig. 1B) induced, after 8 min, the appearance of sporadic bursts with superimposed action potentials, associated with minimal change in baseline at steady state (3 mV) that persisted through the entire application.

Two consecutive applications of oxytocin (1 μM, duration  =  10 min), alternated with 20 min washout phases, were repeated on the same cell (Fig. 1 C) and induced comparable depolarizations (ΔV first application  =  13.34 mV; ΔV last application  =  13.12 mV). Thus, in our experiments, even at higher concentrations, no desensitization appeared when applications of oxytocin were spaced out by at least 20 min.

The average cumulative dose-response curve (Fig. 1 D), obtained from different (3–10) motoneurons, had a shallow slope extending over a 0.5–1000 nM range (EC_50_ of 72 nM). Fig. 1 D also shows that, for each concentration of oxytocin, there was no change in input resistance at rest (filled triangles; one way ANOVA on raw data; P = 0.995; n = 3–4). Hence, these results suggested that the depolarizing effect of oxytocin had a mainly indirect origin. We corroborated this hypothesis by performing experiments in the presence of tetrodotoxin (TTX; 250 nM) to block network synaptic transmission, in analogy with our former study [47]: the example in Fig. 1 E indicates absence of motoneuron depolarization under this condition, without any change in the frequency of miniature post synaptic potentials (1.17±0.24 Hz vs 1.08±0.07 in control TTX solution). Thus, the most likely explanation is that the depolarization mediated by oxytocin was not evoked by the direct action on motoneuron membrane, but it arose from the activation of a premotoneuron network.

### Network effects of oxytocin

**Figure 2:**
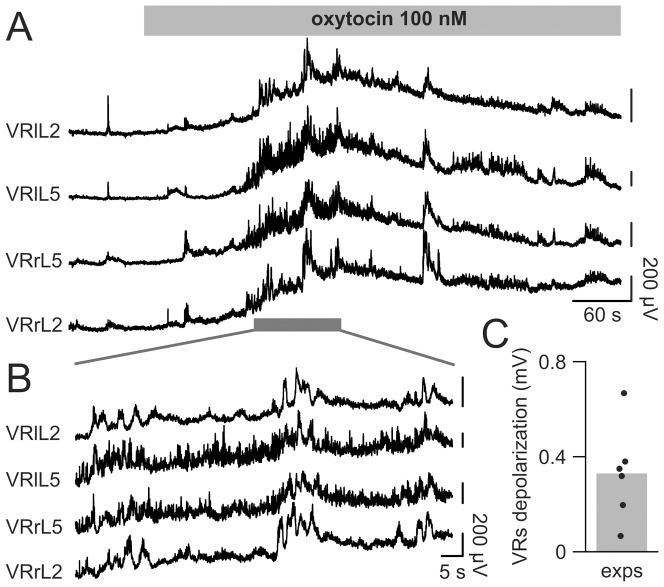
Oxytocin induces VR rhythmic activity. The application of 100(gray bar) depolarizes bilateral VRs at L2 and L5 levels (A). The early phase of VR depolarization is associated with bursts synchronous among all roots, followed by a partial repolarization. B, the rhythmic activity evoked by oxytocin on the four VRs shown in A (gray bar) is displayed, on a faster time scale, to depict synchronous bursts composed of apparently unrelated, fast intraburst oscillations. Histogram (C) illustrates, for each spinal cord, the average depolarization (recorded from 4 VRs) induced by oxytocin 100 nM, while the gray bar shows the mean value of all experiments. All vertical bars are 200 μV.

Extracellular recordings from several VRs expressing the discharge of motoneuron pools and their premotoneuron circuitry (as exemplified in Fig. 2 A) showed strong network activity induced by oxytocin (100 nM) consisting in an initial VR depolarization on both sides of the cord, reaching (after 2 min) a maximal peak of 0.50±0.25 mV (average data from the four recorded VRs) versus the control baseline. After 6 min of continuous application, VR depolarization declined to a stable value (0.31±0.19 mV) versus the control baseline before oxytocin application. During the initial depolarization, irregular VR activity emerged together with bursts (synchronous among all VRs; Fig. 2 B), which eventually faded away.

In a series of six preparations, for each spinal cord, the steady state depolarization induced by 100 nM oxytocin on each VR was recorded and averaged among the four roots to provide the datapoints used to construct the histogram shown in Fig. 2C (0.33±0.20 mV).

**Figure 3:**
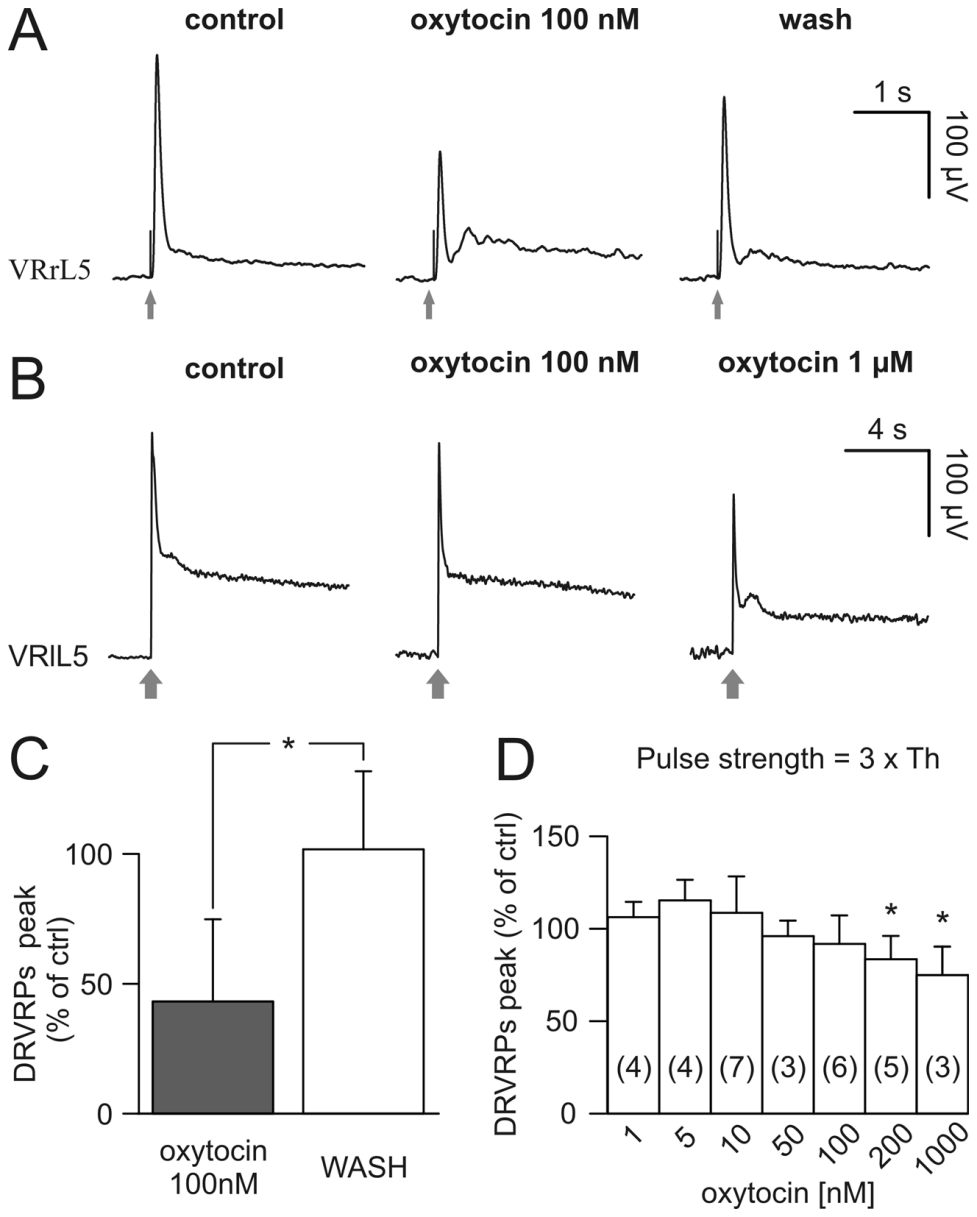
Oxytocin differentially affects DR-VRPs. A, the monosynaptic response elicited by stimulating the homologous DR at just threshold intensity (duration  =  0.1 ms; intensity  =  15 μA, 1×Th) is significantly depressed by the addition of 100 nM of oxytocin, an effect that partially reversed after 20 min washout. B, DR-VRPs, extracellularly recorded from VRlL5, are evoked by strong electrical stimulation of the homologous DR (duration  =  0.1 ms; intensity  =  45 μA, 3×Th; arrows) in control (left), or after applying oxytocin 100 nM (middle) and 1 μM (right). Note records in B are shown on a slower time base to display the secondary component of polysynaptic DR-VRP and, therefore, hide the stimulus artifact. Only the highest tested concentration of the neuropeptide is able to reduce the peak of reflex response. Traces in A and B are mean values from five events and are obtained from different spinal cords. Histograms (C) show summary of responses to low strength of stimulation (1×Th) with significant reduction in the percentage peak obtained from 5 experiments (*; Mann-Whitney rank sum test; P = 0.016). D shows the average percentage variation in peak amplitude of DR-VRPs (with respect to control) evoked by strong stimuli (3×Th), against cumulative increase in oxytocin concentrations. Only the higher concentrations (0.2–1 μM) significantly depress responses (*; Kruskal-Wallis one way ANOVA on ranks followed by multiple comparison vs WASH with Dunn's method; P = 0.004, the number of preparations used to calculate the mean is shown in parentheses; the error bars indicate SD).

We next explored whether the network effects induced by oxytocin were associated to any change in reflex activity. One representative experiment is shown in Fig. 3 A, B in which we averaged five consecutive Dorsal Root-evoked Ventral Root Potentials (DR-VRPs) elicited on one lumbar VR by weak (A) or strong (B) electrical pulses (gray arrows). When weak DR stimuli (intensity  =  10 μA, Th, 1×Th) were used to activate low threshold afferent fibers, 100 nM oxytocin induced a reversible depression of DR-VRPs (Fig. 3 A), as confirmed by the mean values pooled from five spinal cords (Fig. 3 C). On the other hand, at a higher stimulating strength (3×Th), the same oxytocin concentration (100 nM) did not produce any significant change in peak and area of polysynaptic responses (Fig. 3 B middle), while only a 10-fold larger concentration (1 μM) did depress reflexes (Fig. 3 B right). On average, at higher intensities of stimulation, concentrations of oxytocin up to 100 nM did not change DR-VRPs (Fig. 3 D), while a significant decrease (by 20 – 25% vs control) in peak reflex amplitude was detected at 200 nM and 1 μM oxytocin.

### Augmenting concentrations of oxytocin speeded up the disinhibited rhythm by acting selectively on oxytocin receptors

The emergence of irregular bursts during oxytocin application suggested that this neuropeptide could trigger, albeit for a short time, the intrinsic rhythmicity manifested as synchronous discharges from VRs. To further explore this issue, we examined how oxytocin could affect the spontaneous bursting of spinal networks, which appears when spinal inhibition mediated by GABA_A_ and glycine receptors is blocked by strychnine (1 μM) and bicuculline (20 μM) and requires a minimal circuitry restricted to a ventral quadrant of the spinal cord [40].

**Figure 4:**
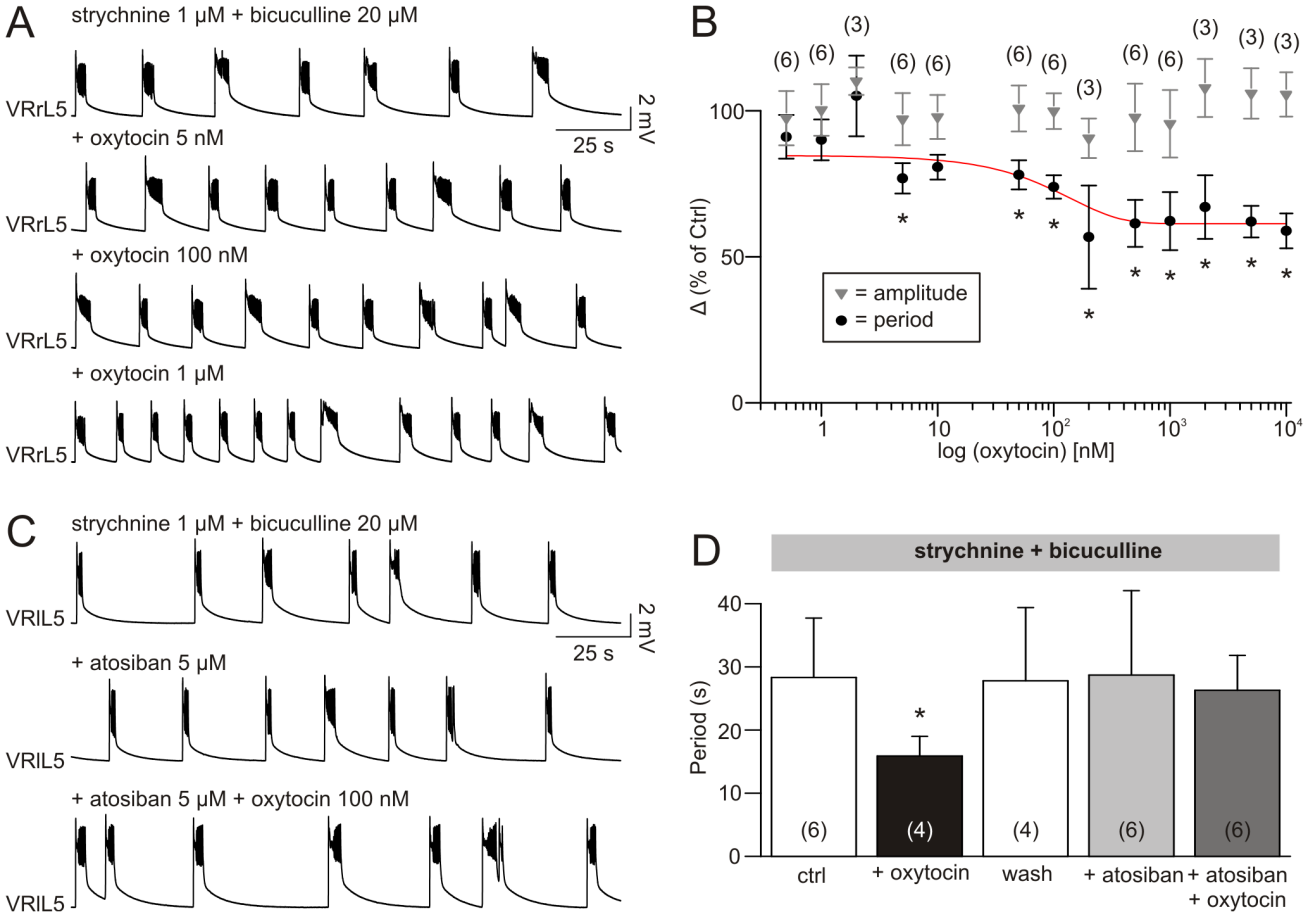
Disinhibited bursting is accelerated by oxytocin, an effect prevented by its selective antagonist. A, regular disinhibited rhythm induced by 1 μM strychnine and 20 μM bicuculline (top trace in A) is stably sped up (with burst length reduction) by the cumulative addition of oxytocin (5, 100 nM and 1 μM), without any further modifications in the characteristics of single bursts. The cumulative dose response curve in B indicates significant reduction in the average period (expressed as a percentage of the mean variation), starting at concentration of 5 nM (*; one way ANOVA followed by multiple comparison vs strychnine + bicuculline only with Dunnett's method; P = <0.001; the number of preparations used to calculate the mean is shown in parentheses; the error bars indicate SD). C, on a different preparation, a stable disinhibited rhythm (top) remains unchanged by the addition of the OTRs antagonist, atosiban (5 μM, middle), which prevents any acceleration during the following addition of oxytocin (100 nM). The histograms in D, which report the average value of period of disinhibited rhythm in correspondence to the different treatments, show significant rhythm acceleration in the presence of oxytocin (100 nM), an effect reverted to control values after washout (30 min), and prevented by the addition of atosiban (5 nM), which does not *per se* vary rhythm periodicity (*; Kruskal-Wallis one way ANOVA on ranks followed by multiple comparison vs strychnine + bicuculline only with Dunn's method; P = 0,038; the number of preparations used to calculate the mean is shown in parentheses; the error bars indicate SD).

In the example of Fig. 4 A, a stable disinhibited rhythm (top trace) was speeded up by 5 nM oxytocin, without changing burst amplitude. On the same preparation, further increases in rhythm frequency were obtained with 100 nM or 1 μM oxytocin. The cumulative dose-response curve in Fig. 4 B reports a dose-dependent reduction in the mean period (expressed as a percent of control) for increasing concentrations of oxytocin (0.5 nM – 10 μM), with an IC_50_ of 55 nM (n = 3–6) with an effect saturation amounting to circa 60% acceleration. Oxytocin did not significantly modify burst amplitude (gray triangles in Fig. 4 B; Kruskal-Wallis one way ANOVA, P = 0.961, n = 3–6) and bursting regularity (calculated as CV value; one way ANOVA, P = 0.300, n = 3–6).

The structure of single disinhibited bursts was altered by the addition of the neuropeptide. In particular, despite an unchanged first phase of depolarizing plateau (one way ANOVA, P = 0.117, n = 9), the total duration of the single burst was reduced (one way ANOVA, P = 0.021, n = 3–6), along with faster intraburst oscillations (one way ANOVA, P = 0.034, n = 3–6), while their number remained unaffected (one way ANOVA, P = 0.765, n = 3–6).

In the presence of strychnine and bicuculline, oxytocin (100 nM) slowly depolarized VRs with a plateau (10 min) of 0.77±0.10 mV (n = 3), which did not statistically differ from the depolarization elicited by the neuropeptide in control conditions (t-test, P = 0.355).

The effects of oxytocin were mediated by OTRs as demonstrated by applying the selective pharmacological antagonist, atosiban (5 μM) that (as shown in Fig. 4 C), without modifying *per se* rhythm features, fully prevented burst acceleration and VR depolarization by 100 nM oxytocin. This observation suggests that OTRs are not endogenously activated during disinhibited rhythm, yet mediate the action of exogenously-applied oxytocin. The histograms in Fig. 4 D summarize the average values of bursting periodicity obtained from 4–6 experiments. While oxytocin (100 nM) significantly reduced the period (black bar), there was no significant variation with atosiban (5 μM) alone or of atosiban plus oxytocin. These results are consistent with an action by oxytocin on spinal networks accessory to the rhythmic ones and capable of modulating intrinsic rhythmicity.

### Neither oxytocin nor atosiban altered frequency and regularity of oscillations of the chemically evoked fictive locomotion

**Figure 5:**
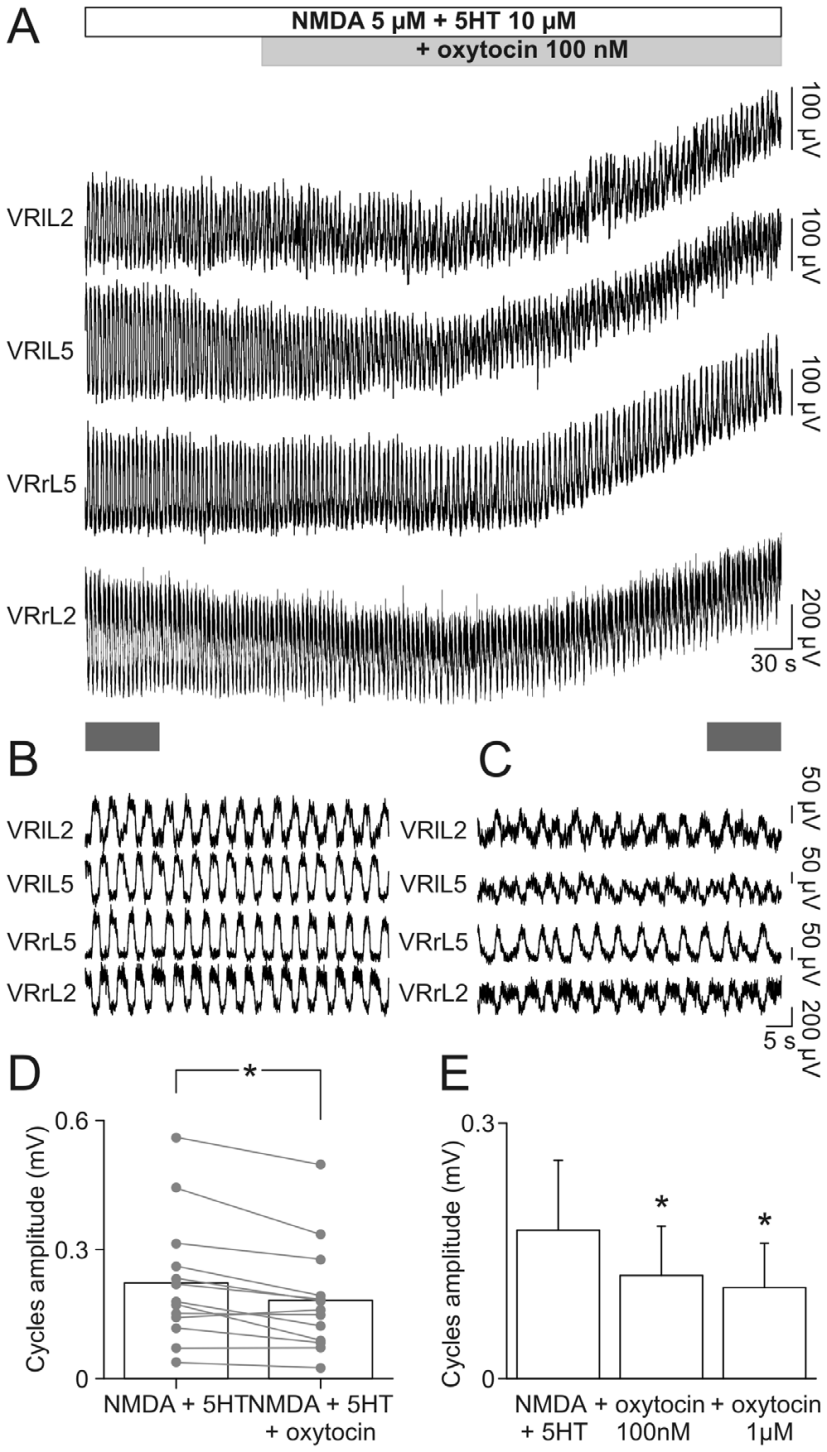
Oxytocin decreases cycle amplitude of FL without changing periodicity. A, a stable FL is induced by the application of 5 μM NMDA and 10 μM 5HT. A sample of traces, corresponding to the gray bar in the bottom-left, shows, on a faster time base, the characteristic double alternation among L2 and L5 homosegmental and homolateral VRs (B). Addition of oxytocin (100 nM; see gray bar in A, top) depolarizes all VRs. As depicted in the insert corresponding to the gray bar in the bottom-right of A, in the presence of oxytocin oscillations maintain their typical double alternation, although with smaller amplitude. The scatter plot in D reports the single values of cycle amplitude for different experiments (gray dots and lines), pointing out the significant reduction in average amplitude of oscillations (open bars) after the addition of oxytocin (0.22±0.15 and 0.18±0.13 mV, respectively; *; paired t-test; P = 0,001; n = 13). Histograms in E depict the reduction in amplitude of FL oscillations in correspondence to cumulative increase in oxytocin concentrations (100 nM and 1 μM) that are equally able to reduce cycle amplitude with respect to control (*, one way repeated measures ANOVA followed by all pairwise multiple comparison procedures with Tukey test, P = 0.004, n = 5).

The discrete effects by oxytocin on spontaneous bursting prompted further experiments to find out if the peptide could modulate locomotor-like oscillations that require a more complex pattern of rhythmic activity including reciprocal inhibition [1]. Fig. 5 A shows (on a slow time base) a stable FL rhythm evoked by 5 μM of NMDA and 10 μM of 5HT (open horizontal bar). On a faster time base, a sample of this record clearly demonstrates the characteristic double alternation of FL cycles recorded from L2 and L5 VRs on both sides of the cord (Fig. 5 B). Addition of 100 nM oxytocin (gray bar in Fig. 5 A) depolarized VRs (0.27±0.06 mV average from four VRs) and reduced the amplitude of locomotor cycles, without modifying frequency or regularity of rhythm (Fig. 5 C). The same observation was repeated with a random sample of 9 experiments, that gave an average depolarization of 0.35±0.22 mV, not statistically different from the depolarization elicited by oxytocin (100 nM) in control conditions (t-test, P = 0.284).

Oxytocin (100 nM) did not modify period (paired t-test, P = 0.054, n = 13) or regularity (Wilcoxon signed rank test, P = 0,094, n = 13) of FL rhythm, while the amplitude of FL oscillations was significantly reduced in each preparation (Fig. 5 D).

Additional experiments were performed in order to verify whether higher doses of oxytocin could vary any of the rhythm parameters unaffected by 100 nM oxytocin. In five spinal cords, on which oxytocin was tested at 100 nM and 1 μM during a stable FL, period or CV values were not statistically different for either concentrations (one way repeated measures ANOVA, P = 0.808 and P = 0.927, respectively), while cycle amplitude was equally reduced by either concentrations with respect to their control (Fig. 5 E; one way repeated measures ANOVA followed by all pairwise multiple comparison procedures with Tukey test, P = 0.004). Finally, as demonstrated with the example of Fig. S1, the oxytocin receptor antagonist atosiban (5 μM) applied together with NMDA (5 μM) and 5HT (10 μM) did not change FL. On average, on 6 spinal cords, the addition of atosiban (5 μM) to a stable FL rhythm induced by NMDA and 5HT did not depolarize VRs (0.20±0.45 mV), nor did it modify period (3.56±1.09 s in ctrl vs. 3.86±0.53 s plus atosiban; paired t-test, P = 0.606), regularity of alternating oscillations (Wilcoxon signed rank test, P = 0.563) or cycle amplitude (Wilcoxon signed rank test, P = 0.688).

### Synergy between oxytocin and NMDA plus 5HT in eliciting FL

**Figure 6:**
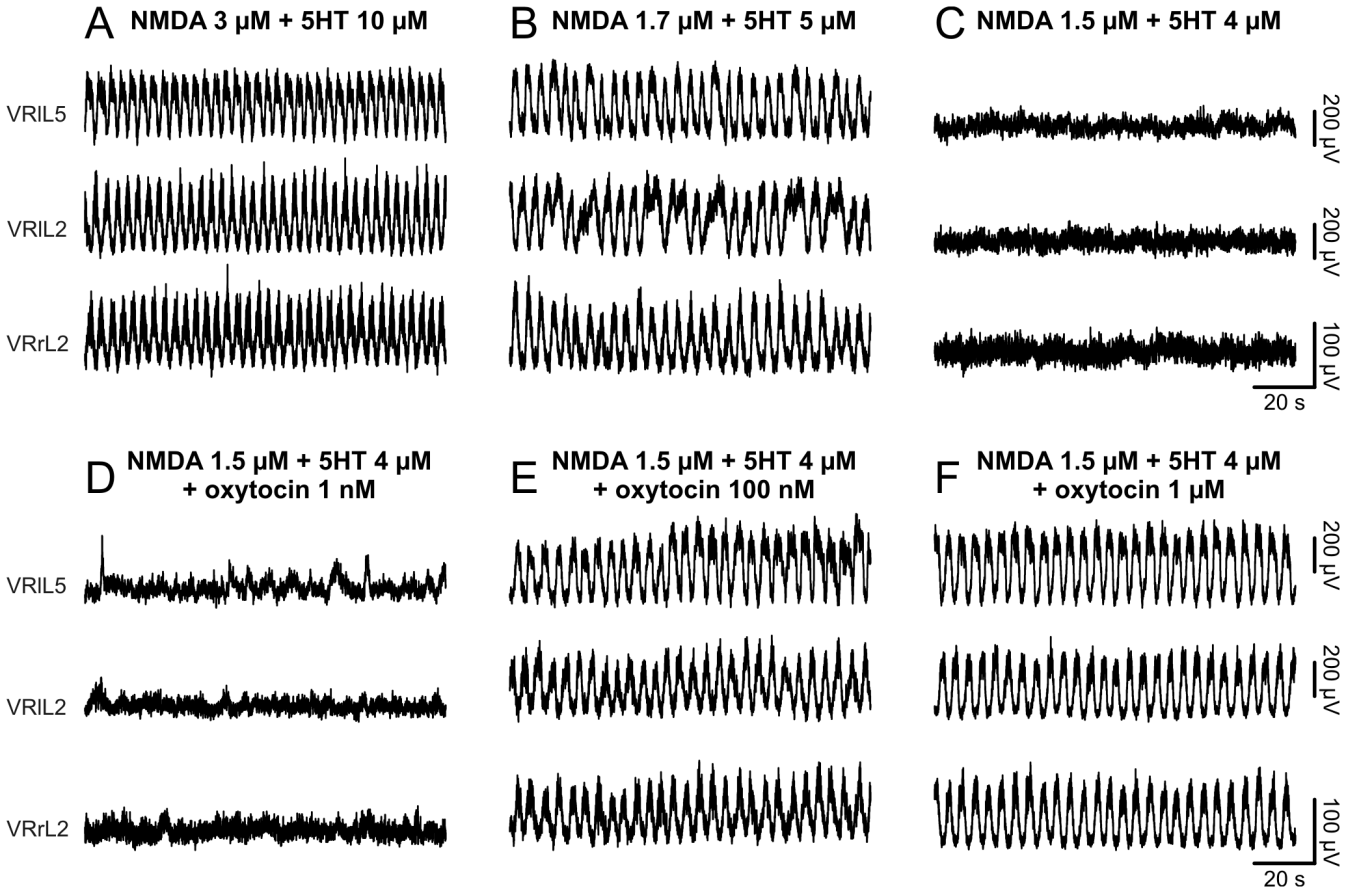
Oxytocin reinstates fictive locomotor oscillations, despite subthreshold concentrations of neurochemicals. Alternating oscillations of a stable FL, evoked by the addition of 3 μM NMDA and 10 μM 5HT (A), slow down once the concentration of neurochemicals is finely titrated down to 1.7 μM NMDA and 5 μM 5HT (B). A further decrease in NMDA (1.5 μM) + 5HT (4 μM) suppresses locomotor-like discharges, which are finally replaced by a tonic activity (C). By adding oxytocin (1 nM) to subthreshold concentrations of NMDA and 5HT, no FL oscillations reappear (D). By augmenting the neuropeptide to 100 nM, locomotor-like oscillations are restored (E). Further increase in oxytocin (1 μM) does not affect periodicity of the reinstated pattern nor its cycle amplitude (F).

Although OTRs did not physiologically control chemically-induced FL, we explored whether the accelerating property shown by this neuropeptide could be exploited to facilitate the activation of locomotor circuits. For this reason a stable FL was first induced using the lowest effective concentrations of NMDA and 5HT (Fig. 6 A). Afterwards, the concentration of neurochemicals was decreased to slow down rhythm period (Fig. 6 B), and eventually to replace FL with a mere irregular activity (Fig. 6 C). Doses of oxytocin as low as 1 nM were not able to re-establish alternating discharges (Fig. 6 D). However, by combining the application of oxytocin (100 nM) with low concentrations of neurochemicals, stable locomotor oscillations reappeared (Fig. 6 E). A further increase in oxytocin concentrations did not modify the features of the reinstated FL (Fig. 6 F).

Similar experiments were repeated with 12 preparations, in which 100 nM oxytocin in the presence of subthreshold concentrations of neurochemicals induced a FL similar to control for mean period, period CV and cycle amplitude (Table S1 summarizes these data). To explore the possibility that an increase in oxytocin might change any of the parameters related to the rhythm rescued by 100 nM oxytocin, we cumulatively added 1 μM oxytocin to subthreshold concentrations of neurochemicals. On average, neither periodicity (one way repeated measures ANOVA, P = 0.313, n = 3), regularity (one way repeated measures ANOVA, P = 0.232, n = 3) nor cycle amplitude (one way repeated measures ANOVA, P = 0.296, n = 3) were further affected. Finally, in five experiments, oxytocin applied within the concentration of 1–50 nM was unable to activate a subthreshold FL.

### Oxytocin facilitation of fictive locomotor patterns requires endogenous 5HT synthesis

**Figure 7:**
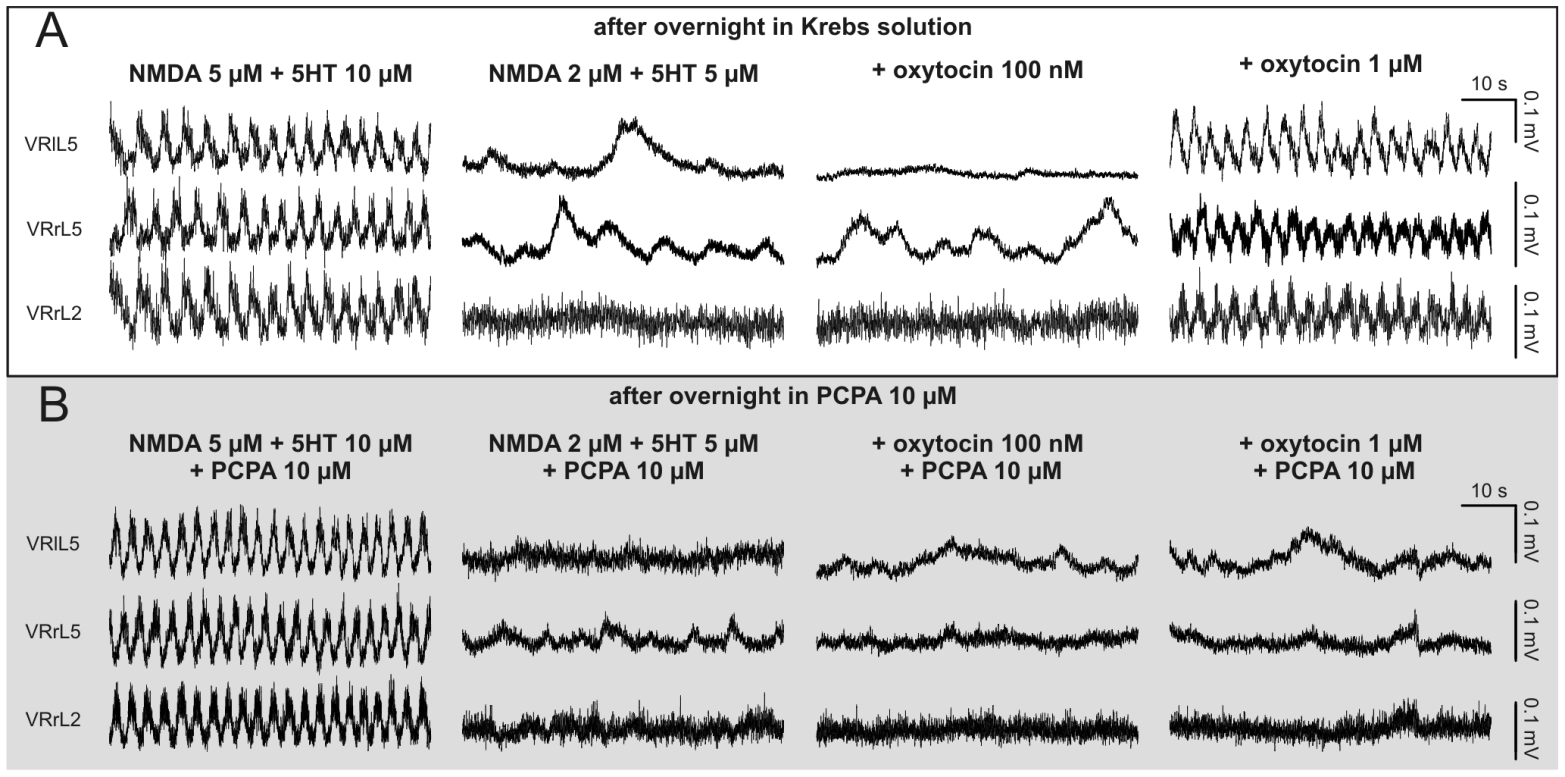
Inhibitor of endogenous 5HT synthesis prevents facilitation by oxytocin of FL. Two different preparations are maintained overnight in Krebs solution, upper traces (A), or in the presence of the inhibitor of 5HT synthesis (PCPA, 10 μM; lower traces, light gray field, B), respectively. On the following day, in both preparations, a stable FL is recorded in the presence of NMDA (5 μM) plus 5HT (10 μM; A, B, left). By decreasing the concentration of neurochemicals, alternating oscillations are replaced by a tonic activity with slow depolarizing events, apparently unrelated among different VRs (A, B, second panels). The addition of oxytocin (100 nM) to subthreshold concentrations of NMDA and 5HT fails to reinstate FL (A, B, third panels). However, further increase in oxytocin to 1 μM induces reappearance of a stable FL only in the preparation maintained in Krebs solution (A, right), while, in the one incubated with PCPA, no alternating oscillations are observed (B, right). Note that PCPA (10 μM) is continuously perfused during all different experimental phases conducted on the spinal cords treated in PCPA overnight (B).

Since OTRs are reported to modulate the release of 5HT [50], [51] that largely contributes to FL [52], it seemed likely that oxytocin-mediated increase in endogenous 5HT release contributed to bring FL patterns to threshold. To explore this hypothesis, we performed experiments in which the isolated spinal cord was treated overnight with the inhibitor of the tryptophan hydroxylase, p-chlorophenylalanine (PCPA, 10 μM), with the aim of reducing the synthesis of endogenous 5HT. Fig. 7 shows two typical experiments run in parallel, in which two different spinal cords were kept overnight in a Krebs (A) or PCPA (10 μM; B) solution, respectively. On the second day, in the presence of NMDA (5 μM) and 5HT (10 μM), both preparations displayed a stable FL (Fig. 7 A,B, left panels). On average, all cords tested the day after, showed a FL period of 2.98±1.22 s (CV = 0.12±0.04; n = 15), without any significant difference between the sham group and the one treated with PCPA (t-test, P = 0.843, n = 6–9).

After washout (20 min), preparations were first perfused with subthreshold concentrations of neurochemicals (Fig. 7 A,B, second panels) to which 100 nM oxytocin was subsequently added (Fig. 7 A,B) without emergence of any FL. In the example depicted in Fig. 7 A (right), further increase in oxytocin concentration (1 μM) triggered a stable FL similar to control for period (90% of control), CV (0.12 vs 0.15 in control) and amplitude (90% of control) values. This observation was replicated in three out of four sham spinal cords, as 1 μM oxytocin re-established the FL rhythm with regularity (CV period  =  0.12), period (paired t-test, P = 0.266, n = 3) and amplitude (paired t-test, P = 0.669, n = 3) comparable to those induced on the same preparations by NMDA (5 μM) and 5HT (10 μM). On the contrary, in all preparations treated with PCPA (10 μM), as exemplified in Fig. 7 B, oxytocin at 100 nM (9/9) or 1 μM (7/7) concentration never triggered FL in the presence of subthreshold NMDA and 5HT, even if these preparations displayed control patterns with NMDA (5 μM) plus 5HT (10 μM).

### Nanomolar concentrations of oxytocin synergize with the delivery of innovative protocols of low intensity electrical stimulation

The novel FL*i*stim protocol of DR electrical stimulation based on capturing the FL cycles and applying them to one DR, has been shown to optimally activate the *in vitro* CPG much more effectively than standard trains of DR pulses: the resulting rhythm shows, however, stereotypic periodicity [37].

**Figure 8:**
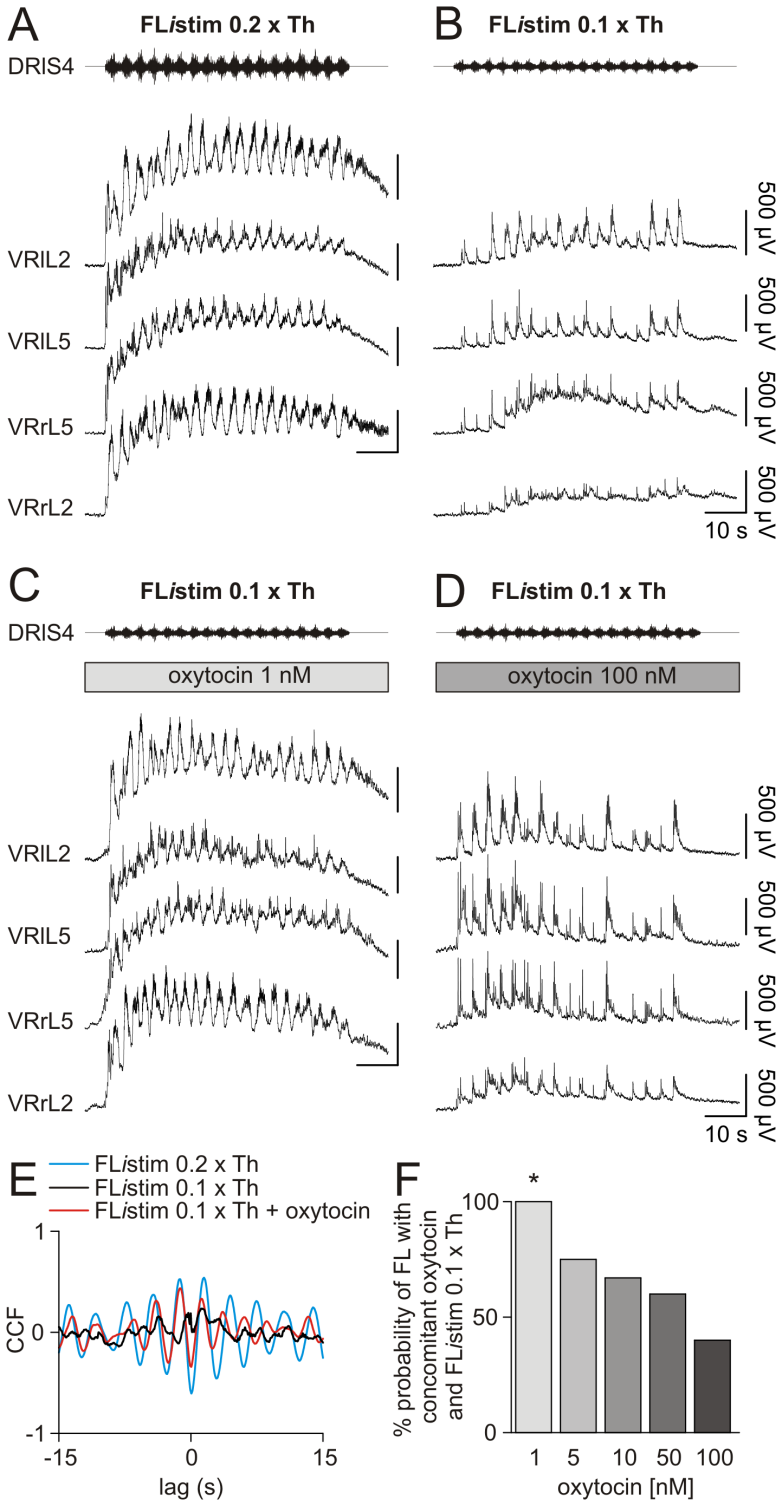
Low nanomolar concentrations of oxytocin synergize with FL*i*stim in expressing fictive locomotor patterns. A, FL*i*stim (0.2×Th) generates cumulative depolarization with alternating oscillations among homolateral L2 and L5 VRs and among controlateral homosegmental VRs. When stimulus intensity is halved (0.1×Th), a slight cumulative depolarization with synchronous discharges (time locked with the stimulating pattern) appears among all VRs (B). Despite the weak electrical stimulation (FL*i*stim 0.1×Th), the addition of low concentration of oxytocin (1 nM) re-establishes cumulative depolarization and FL cycles (C). Increased concentration of oxytocin (100 nM) fails to synergize with the same FL*i*stim 0.1×Th (D). Cross correlation analysis for traces related to the pair of L2 VRs in A–C shows a negative peak centered around zero lag for suprathreshold FL*i*stim (blue trace) or subthreshold FL*i*stim plus 1 nM oxytocin (red trace). The weak FL*i*stim alone (black trace) has a CCF value close to zero, corresponding to an uncorrelated activity among the two VRs (E). F, histograms show that, by increasing the concentration of oxytocin, the probability of bringing FL to threshold with a weak FL*i*stim diminishes in a dose dependent manner (*; Chi-square vs FL*i*stim 0.1×Th alone; P = 0.005; n = 8).

The present study investigated whether oxytocin could synergize with a subthreshold FL*i*stim. Fig. 8 shows one example taken from the same preparation tested with various protocols. Thus, the FL*i*stim protocol (duration  =  60 s, intensity  =  0.2×Th) [37], [39] was first applied to a sacral DR, inducing a cumulative depolarization with superimposed an episode of alternating cycles on L2 and L5 VRs (on both sides), lasting throughout the delivery of the stimulating pattern (Fig. 8 A). At the same strength of stimulation, alternating oscillations evoked by FL*i*stim were not statistically modified by the conjoint application of oxytocin (1 – 100 nM; not shown).

By lowering the strength of FL*i*stim (0.1×Th), the resulting cumulative depolarization was smaller with sporadic oscillations, synchronous among all VRs (Fig. 8 B). Nevertheless, applying the lowest tested concentration of oxytocin (1 nM), the weak FL*i*stim was now able to evoke a cumulative depolarization (0.97 mV) with a superimposed episode of FL (Fig. 8 C), comparable to the one recorded in control with FL*i*stim delivered at optimal amplitude (0.2×Th; Fig. 8 A). However, increasing oxytocin up to 100 nM (to induce 0.96 mV cumulative depolarization *per se*) together with FL*i*stim at 0.1×Th failed to elicit a FL (Fig. 8 D).

The cross-correlograms on the pair of homosegmental VRs at L2 level for the three protocols are superimposed in Fig. 8 E to assess cycle alternation. Thus, the negative peak centred around zero lag during the coapplication of 0.1×Th FL*i*stim plus oxytocin 1 nM (red trace) confirms FL pattern comparable to the one evoked by FL*i*stim at 0.2×Th (blue trace), while the plot for 0.1×Th FL*i*stim alone yielded a flat trace (black), indicating uncorrelated activity.

In eight experiments, oxytocin (1 nM) in combination with a weak FL*i*stim, always triggered the onset of FL as much as a stronger FL*i*stim did. Conversely, the probability to induce FL fell with stepwise increase in oxytocin concentrations, while keeping the same weak FL*i*stim (Fig. 8 F) without variation in the amplitude of cumulative depolarization (Kruskal Wallis one way ANOVA on ranks followed by post hoc analysis with Dunn's method, P = 0.286; n = 8).

One component of the oxytocin complex action on FL*i*stim might have comprised dose-dependent modulation of pre-synaptic inhibition on afferent inputs investigated as reported by Hochman et al. [53]. Thus, experiments were performed in which dorsal root potentials were recorded in response to electrical stimulation of the adjacent dorsal root (DR-DRPs), at both low and high pulse strength (Fig. S2 A, B). In the presence of oxytocin (1 or 100 nM), no differences were observed in the peak amplitude of DR-DRPs, at either low (Fig. S2 C) or high (Fig. S2 D) intensity, or in the area recorded with the high-strength stimulation (Fig. S2 E). These results show that the enhancement in post-synaptic responses generated by the downstream motor networks after application of oxytocin, was not accompanied by any detectable modulation of pre-synaptic inhibition on primary afferent signals.

## Discussion

The present study analyses the complex effects evoked by oxytocin in modulating the basal characteristics of single motoneurons, the synaptic responses induced by afferent stimulation, and two different types of locomotor network activity. Although these data were collected from the *in vitro* mammal spinal cord, they can help to interpret the functional impact of oxytocin targets on spinal circuits and emphasize how even nanomolar concentrations of this neuropeptide could synergize with innovative stimulation protocols to elicit locomotor network activation.

### Facilitatory effects by oxytocin

While application of oxytocin *per se* never elicited FL in line with previous observations [32], [33], the peptide did evoke a number of responses that ranged from dose-related motoneuron depolarization (lacking change in input resistance) with repetitive or burst firing, VR depolarization associated with synchronous discharges, and acceleration of disinhibited bursting (with burst length reduction). All these effects were persistent and showed no tachyphylaxis. When network activity was blocked by TTX to minimize spike-dependent neurotransmission [54], inhibition of oxytocin responses was observed in accordance with a previous study [55]. In slices of neonatal rat spinal cords, no depolarization of motoneurons is observed, indicating that a substantial multisegmental network is a prerequisite for observing these responses [23], [56]. These results suggest that most (if not all) of these actions were likely exerted at premotoneuron level. This notion is consistent with the description of sparse oxytocin-containing fibers contacting motoneurons [57] and lack of evidence supporting the expression of OTRs by motoneurons [23], [26], [27], [58]. Despite the report of a subpopulation of glycinergic dorsal interneurons with OTR [23], the present data obtained with strychnine and bicuculline application suggest that the activation of such interneurons is not mandatory to produce these stimulatory effects. Future studies are necessary to identify the precise premotoneuron elements responsible for the observed effects by oxytocin in analogy with the approach used to dissect out the mechanism of action of dopamine to stabilize FL and excite motoneurons [59].

### Oxytocin-mediated modulation of synaptic responses

Unlike the facilitatory effects produced by oxytocin on the basal activity of spinal circuits, oxytocin elicited more complex responses when such spinal networks were electrically or chemically stimulated. Although low nanomolar concentrations of the peptide had no significant action on reflexes, at concentrations ≥ 100 nM oxytocin significantly depressed DR-VRPs (see also [60]), especially those evoked by weak stimuli. Since OTRs are not expressed by afferent fibers [61], the observed changes in synaptic transmission were probably generated within spinal networks. Assuming that one important factor determining the size of the DR-VRP is the extent of the activated premotoneuron circuitry in turn related to the electrical pulse strength, the present results suggests that the depressant action of oxytocin was dependent on the activation state of network elements. This suggestion was further explored by studying how a function-related network rhythm of the CPG was modulated by the peptide.

### Oxytocin facilitates the expression of the locomotor pattern

Despite its inhibitory effects on reflex activity, oxytocin (≥ 100 nM) showed functional synergy with NMDA and 5HT in triggering oscillations when the CPG activity was subthreshold [32], [33]. When locomotor networks were fully activated by NMDA and 5HT to express strong neuronal discharges alternating among distributed motor pools [62], 100 nM oxytocin unexpectedly failed to up or downregulate FL, suggesting that either any reflex depression was restricted to certain pathways not essential for FL (albeit impinging on motoneurons), and/or that the degree of neuronal activation by NMDA and 5HT was large enough to overwhelm oxytocin-mediated decrease in synaptic transmission. This view is consistent with the lack of effects by the OTR antagonist atosiban on a stable FL pattern.

The precise mechanism responsible for these divergent effects by oxytocin remains unclear. Nevertheless, similar effects were also observed with extracellular recordings from rat dorsal horn neurons in vivo with half of them being inhibited and the rest being activated by focally applied oxytocin, suggesting activation of inhibitory interneurons upstream of excitatory neurons [63]. While these in vivo results preclude the possibility of in vitro artefacts, various hypotheses might be advanced to account for the action of oxytocin on locomotor networks. In fact, although the spinal locomotor networks include distributed neuronal elements as indicated by functional labelling experiments [64], the expression of OTRs by lamina X neurons [27], that comprise commissural cells suitable to generate adaptable inputs to fine tune locomotor outputs [65], appears to link the effects of oxytocin to the pattern-generating networks.

A potential mechanism for the facilitatory role played by oxytocin might reside in the reported enhancement of endogenous 5HT release [50], [51] because this biogenic amine is well-known to potently modulate locomotor-like activity [52]. To test this notion in the present study, 5HT synthesis was inhibited by overnight incubation with PCPA [44] and fully prevented any facilitatory action by oxytocin on the locomotor CPG. Nevertheless, because 5HT receptors include a large family of subtypes [66] with multiple effects and even functionally-distinct targets in the rat spinal cord [67], [68], [69], it is proposed that the functional outcome of the oxytocin action might be related to where and how extensively endogenous 5HT was concurrently liberated. In addition, as oxytocin positively modulates AMPA receptor-dependent transmission in a subpopulation of neurons only [70], location and synaptic contact topography of such neurons may determine the expression of oxytocin action.

A further possibility to account for the multifarious effects by oxytocin relies on the peculiar characteristics of its G-protein coupled receptors that can be coupled to different G-proteins (activating divergent intracellular signalling pathways). These receptors are often promiscuous, as a single receptor subtype may couple to more than one G-protein, thus activating, in the same cells, multiple responses at the same time [71], [72]. Moreover, OTR may cluster together to produce functional oligodimers whose assembly and signalling strength depends on the agonist concentration with differential results in terms of functional responses [71], [73]. Through a combination of these properties, oxytocin may show different potency/efficacy via different signalling pathways activated by the same receptor, in analogy with a multistate model of receptor activation [72].

### Oxytocin synergizes with stimulation by weak noisy waveforms

There were several analogies in the effects by oxytocin on FL evoked by chemical agents or by FL*i*stim. In the present experiments, oxytocin facilitated the emergence of FL in the presence of a train of weak FL*i*stim *per se* unable to elicit persistent alternating patterns. The probability of triggering FL depended on the oxytocin concentration, i.e. when it was low, a high probability of success emerged, conversely with high concentrations it was more difficult to induce FL. It is noteworthy that the most favourable outcome therefore implied weak FL*i*stim and low oxytocin, a paradigm that, at least in theory if applied in vivo, should be the less prone to evoke unwanted side effects cause by peripheral action of oxytocin or local dysfunction by application of strong current pulses. The only apparent difference of this paradigm against FL caused by NMDA and 5HT was the effective concentration range of oxytocin that was optimal at small doses found to be ineffective on chemically-induced FL. It should be borne in mind that FL*i*stim requires repeated activation of dorsal afferents and that any reflex-depressant action by larger doses of oxytocin would probably have a negative impact on efficient signalling to attain CPG activation. This problem would be circumvented by chemical FL as afferent fibres are not concomitantly stimulated because the locomotor CPG displays a modular organization, whereby different inputs may activate subpopulations of interneurons, that only partially overlap [62], [74].

The synergy between weak FL*i*stim and low oxytocin indicated an interesting protocol whereby FL*i*stim, due to the low stimulation intensities used, might represent a signal to activate only a network subpopulation crucial for the expression of FL, unlike the more generalized activation obtained with neurochemicals, which inevitably recruits many other spinal interneurons, some of which even unrelated to locomotion [62]. The most parsimonious hypothesis of the observed synergy is that a discrete distribution of OTRs with intrinsic properties (like affinity or intracellular effector coupling) might be found on certain network elements that selectively contribute to the expression of FL.

## Conclusions

Due to the well defined sensory input through DRs and motor output through VRs, the isolated neonatal rodent spinal cord represents a suitable model to investigate innovative protocols of afferent stimulation able to optimally activate the spinal interneuronal network [75]. The combination of intra and extracellular recordings, associated with the direct application of selective pharmacological protocols for inducing different types of rhythmic activity, allowed us to postulate that OTRs are strategically located on locomotor circuit nodes whose activation is necessary to propagate and recruit the CPG operation.

Current interest in the central effects of oxytocin includes clinical trials of this peptide for schizophrenia or learning disorders [25]. The wide gap between these disorders points a broad role of the neuropeptide in modulating central networks and its overall safety in man. Thus, the present data may add a further hint to test low doses of oxytocin in combination with direct electrical stimulation of the spinal cord [5], [76] in exploiting the residual locomotor capacities after spinal damage. In conclusion, our results appear interesting when considering ongoing clinical trials targeting oxytocin for spinal cord dysfunction (http://clinicaltrials.gov).

## Supporting Information

Figure S1 Endogenous oxytocin does not modulate locomotor patterns. A, a stable FL is recorded in response to the co-application of NMDA (5 μM) and 5HT (10 μM). The addition of the selective antagonist for OTRs (atosiban, 5 μM) does not alter periodicity of FL rhythm or amplitude of oscillations.
(TIF)

Figure S2 DR-DRPs are unaffected by oxytocin. Depolarizing potentials are recorded from DRlL5 following electric stimulation of the controlateral DR by a series of single pulses (duration  =  0.1 ms, 0.016 Hz). A, average DR-DRP evoked by low- strength stimulation (1×Th) is unchanged by increasing concentrations of oxytocin (1 nM, middle; 100 nM, right). Note the artefact of stimulation as indicated by arrows. On the same preparation, by augmenting the pulse strength (delivered as indicated by the arrows) to evoke larger and longer DRPs, no significant change is induced by this neuropeptide (B). Traces in A and B are mean of five responses. Histograms for the mean values obtained from four experiments, demonstrate that the addition of oxytocin, at both 100 nM and 1 μM, does not alter peak of DR-DRPs evoked at lower strength (C; one way repeated measures ANOVA, P = 0.155, n = 4), or peak (D; one way repeated measures ANOVA, P = 0.392, n = 4) and area (E; one way repeated measures ANOVA, P = 0.306, n = 4) of responses at the higher strength of stimulation.
(TIF)

Table S1 Characteristics of FL patterns induced by NMDA + 5HT or in the presence of subthreshold concentrations of neurochemicals + oxytocin.
(DOC)
