# Supplementary figures and images for: Nanomolar Oxytocin Synergizes with Weak Electrical Afferent Stimulation to Activate the Locomotor CPG of the Rat Spinal Cord In Vitro
Source: PLoS One. 2014 Mar 21;9(3):e92967. doi: 10.1371/journal.pone.0092967 (PMC3962494; doi:10.1371/journal.pone.0092967)

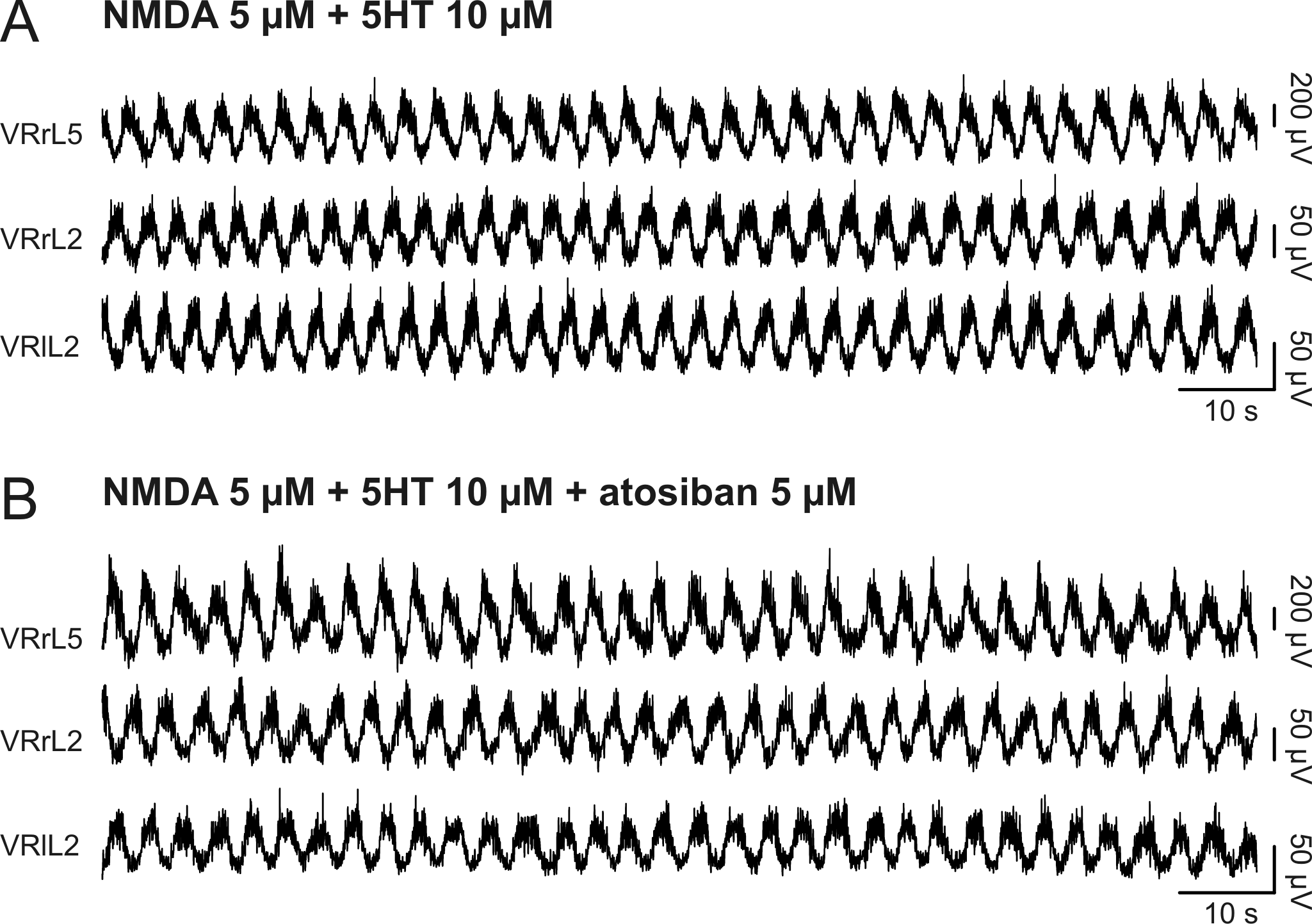

Supplement: Figure S1 — Endogenous oxytocin does not modulate locomotor patterns. A, a stable FL is recorded in response to the co-application of NMDA (5 μM) and 5HT (10 μM). The addition of the selective antagonist for OTRs (atosiban, 5 μM) does not alter periodicity of FL rhythm or amplitude of oscillations. (TIF) [file pone.0092967.s001.tif]

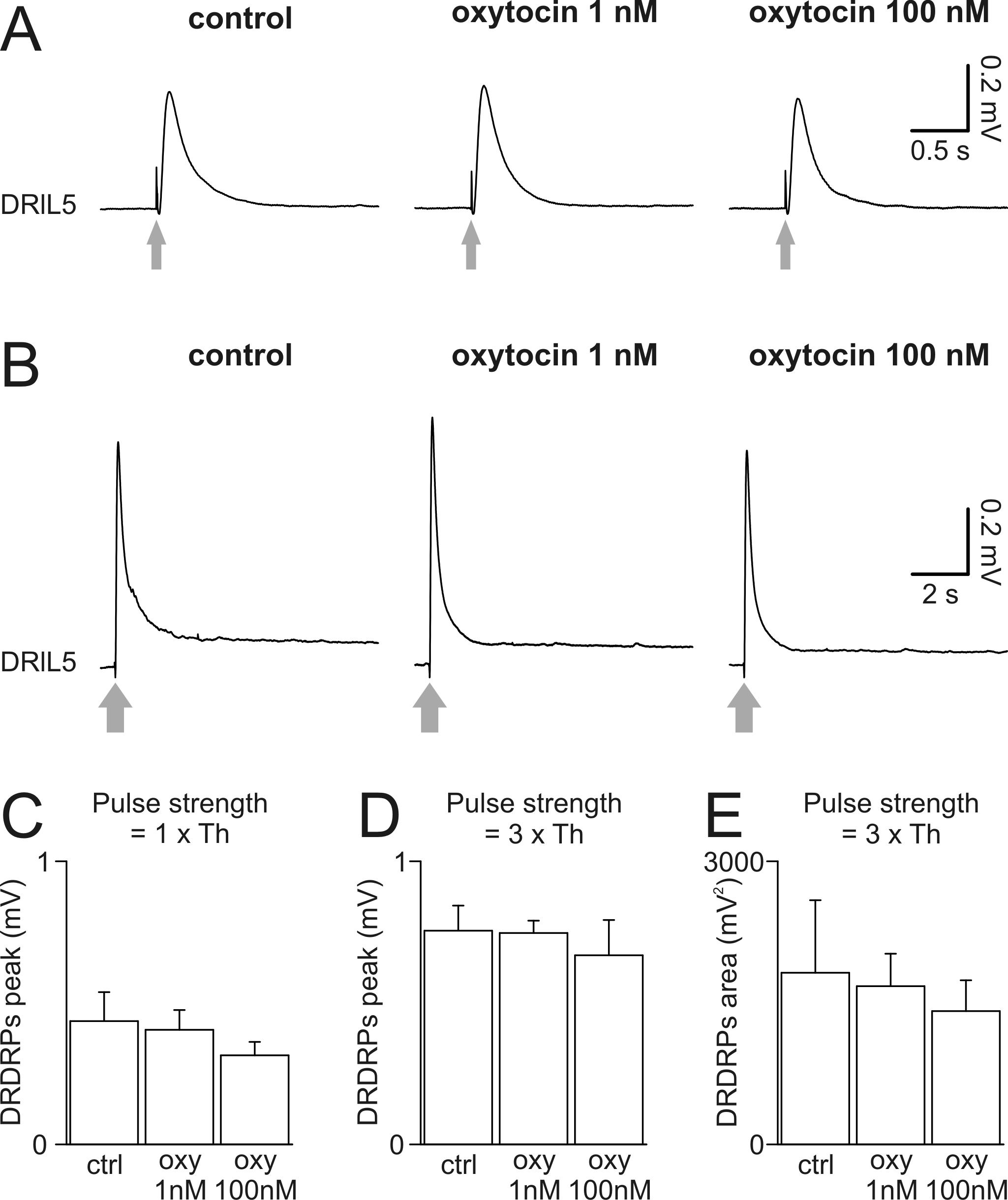

Supplement: Figure S2 — DR-DRPs are unaffected by oxytocin. Depolarizing potentials are recorded from DRlL5 following electric stimulation of the controlateral DR by a series of single pulses (duration = 0.1 ms, 0.016 Hz). A, average DR-DRP evoked by low- strength stimulation (1×Th) is unchanged by increasing concentrations of oxytocin (1 nM, middle; 100 nM, right). Note the artefact of stimulation as indicated by arrows. On the same preparation, by augmenting the pulse strength (delivered as indicated by the arrows) to evoke larger and longer DRPs, no significant change is induced by this neuropeptide (B). Traces in A and B are mean of five responses. Histograms for the mean values obtained from four experiments, demonstrate that the addition of oxytocin, at both 100 nM and 1 μM, does not alter peak of DR-DRPs evoked at lower strength (C; one way repeated measures ANOVA, P = 0.155, n = 4), or peak (D; one way repeated measures ANOVA, P = 0.392, n = 4) and area (E; one way repeated measures ANOVA, P = 0.306, n = 4) of responses at the higher strength of stimulation. (TIF) [file pone.0092967.s002.tif]
