# Supplementary material for: Nanomolar Oxytocin Synergizes with Weak Electrical Afferent Stimulation to Activate the Locomotor CPG of the Rat Spinal Cord In Vitro
Source: PLoS One. 2014 Mar 21;9(3):e92967. doi: 10.1371/journal.pone.0092967 (PMC3962494; doi:10.1371/journal.pone.0092967)
Supplement: Table S1 — Characteristics of FL patterns induced by NMDA + 5HT or in the presence of subthreshold concentrations of neurochemicals + oxytocin. (DOC) [file pone.0092967.s003.doc]

**Table S1. Characteristics of FL patterns induced by NMDA + 5HT or in the presence of subthreshold concentrations of neurochemicals + oxytocin**

|  | **NMDA**  **+ 5HT** | **Subthreshold NMDA and 5HT + oxytocin (100 nM)** | **Statistical test**  **(n = 12 cords)** |
| --- | --- | --- | --- |
| **Period (s)**  **± SD** | 4.26 **±**  1.00 | 5.52 **±**  2.23 | paired t-test,  P = 0.085 |
| **CV of Period**  **± SD** | 0.13 **±**  0.10 | 0.13 **±**  0.08 | Wilcoxon signed rank test,  P = 0.733 |
| **Amplitude (mV)**  **± SD** | 0.24 **±**  0.15 | 0.24 **±**  0.21 | paired t-test,  P = 0.339 |
